# Genetic sleep deprivation: using sleep mutants to study sleep functions

**DOI:** 10.15252/embr.201846807

**Published:** 2019-02-25

**Authors:** Henrik Bringmann

**Affiliations:** ^1^ Max Planck Institute for Biophysical Chemistry Göttingen Germany

**Keywords:** genetics, model organism, optogenetics, sleep, sleep deprivation, Chromatin, Epigenetics, Genomics & Functional Genomics, Molecular Biology of Disease, Neuroscience

## Abstract

Sleep is a fundamental conserved physiological state in animals and humans. It may serve multiple functions, ranging from energy conservation to higher brain operation. Understanding sleep functions and the underlying mechanisms requires the study of sleeplessness and its consequences. The traditional approach to remove sleep is sleep deprivation (SD) by sensory stimulation. However, stimulation‐induced SD can be stressful and can cause non‐specific side effects. An emerging alternative method is “genetic SD”, which removes sleep using genetics or optogenetics. Sleep requires sleep‐active neurons and their regulators. Thus, genetic impairment of sleep circuits might lead to more specific and comprehensive sleep loss. Here, I discuss the advantages and limits of genetic SD in key genetic sleep model animals: rodents, zebrafish, fruit flies and roundworms, and how the study of genetic SD alters our view of sleep functions. Genetic SD typically causes less severe phenotypes compared with stimulation‐induced SD, suggesting that sensory stimulation‐induced SD may have overestimated the role of sleep, calling for a re‐investigation of sleep functions.

**Glossary**
ALA: name of a specific *Caenorhabditis elegans* interneuron/mechanosensory neuron
AMPA: α‐amino‐3‐hydroxy‐5‐methyl‐4‐isoxazolepropionic acid
CNO: clozapine‐N‐oxide
dFB: dorsal fan‐shaped body
EEG: electroencephalogram
EGF: epidermal growth factor
GABA: γ‐aminobutyric acid
GPCR: G protein‐coupled receptor
HPA: hypothalamic–pituitary–adrenal axis
MS: medial septum
PB: parabrachial nuclei
PI: pars intercerebralis
PZ: parafacial zone
REM: rapid eye movement
RIS: ring interneuron s, name of a specific *C. elegans* interneuron
ROS: reactive oxygen species
SD: sleep deprivation
SIK3: salt‐inducible kinase 3
VLPO: ventrolateral preoptic nucleus

## Introduction

We spend about one‐third of our lives asleep. Sleep curtailment is detrimental to human health and is associated with an increased risk of infection, depression, cardiovascular disease, obesity, type 2 diabetes, and cancer, illustrating the importance of this time investment. The high prevalence of insomnia and insufficient sleep quality in modern societies thus presents a massive unmet health problem 1, 2, 3, 4. Sleep appears to affect virtually all aspects of animal physiology, and numerous processes have been proposed to depend on the regular occurrence of sleep. While the broad detrimental consequences of sleep loss are obvious, there still is no consensus as to what the direct consequences of sleep loss are, and how sleep carries out its functions at the molecular level.

Sleep research first focused on humans. Seminal work using EEG recordings showed that humans have two types of sleep, REM sleep and non‐REM sleep, which are also called active and quiet sleep, respectively. During REM sleep, the brain shows high asynchronous activity similar to wake, concomitant with paralysis of striated muscles, with a few exceptions including the musculature controlling eye movement or breathing. During non‐REM sleep, both muscles and neurons show reduced activity with highly synchronous brain activity 5, 6. Using the electrophysiological characteristics of human sleep, it has been possible to detect both types of sleep in a wide range of mammals and birds 7, 8. Even reptiles possess two different states of sleep that resemble non‐REM and REM sleep, whereas amphibians appear to only show quiet sleep 9. This led to the conclusion that sleep diverged at the base of the amniotes into non‐REM and REM sleep.

Behavioral quiescence has long been observed across species, including invertebrates. However, defining sleep using EEG correlates for REM and non‐REM sleep often is not possible due to brain anatomical differences. Nevertheless, quiescence can also be identified as sleep by using four key behavioral criteria 10. (i) A typical posture is assumed that is compatible with reduced muscle activity. (ii) Sleep reduces responsiveness to sensory stimulation, indicating a global neural dampening that extends to sensory systems, and which contrasts sleep to quiet wakefulness. (iii) Sleep is rapidly reversible, meaning that the human or animal can be readily awoken, separating sleep from coma or paralysis. (iv) Sleep is under homeostatic regulation, implying that mechanisms exist that ensure that this state takes place, underscoring its importance 10. By applying these behavioral criteria, sleep has been identified in all animals that have a nervous system, with cnidaria presenting the most basal phylum in which sleep has been detected 11. Thus, sleep is much more widespread among animals than initially thought 12. Particularly important was the identification of sleep in three key non‐mammalian animal models, zebrafish, *Drosophila*, and *Caenorhabditis elegans*, as these models facilitate a molecular and mechanistic dissection of sleep 13, 14, 15, 16, 17.

**Figure 1:**
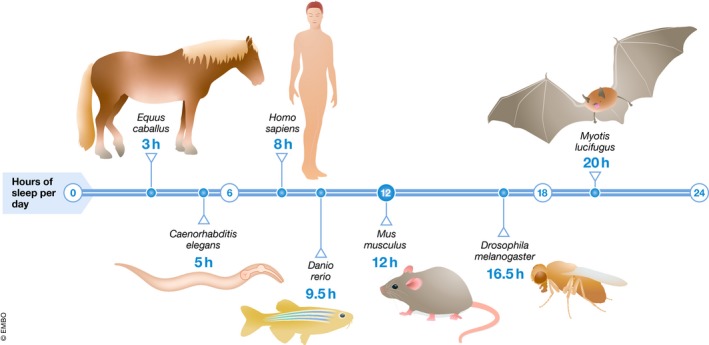
Sleep time fraction varies greatly but does not drop below 10% Sleep time fraction varies between 3–20 h/24 h with large herbivores sleeping little and bats sleeping a lot 21. Model organisms fall within the range of wild species 38, 85, 103, 124.

Compelling evidence for the existence of a species that has a nervous system but never sleeps is lacking, but the amount of sleep is highly plastic and some animals can get away with little sleep. Environmental conditions impact sleep need, and the time spent in sleep differs substantially across species. Under extreme conditions, temporary sleep restriction or even complete loss appears to exist and confers a selective advantage. For example, migrating and mating birds appear to be able to suspend or reduce the need to sleep for at least several days 18, 19. Also, some species, such as large herbivores or cave‐dwelling fish, manage to live with sleeping only little, and even 3 h per day can be sufficient 20, 21. On the other extreme, some animals such as bats sleep up to 20 h per day 21. This suggests that the amount of sleep is adapted to, and depends on ecological constraints, perhaps to regulate behavior and to preserve energy 22. Because animals appear to be asleep for at least 10% of their time, a lower limit of how little sleep is required for survival seems to exist (Fig 1).

## Functions and molecular underpinnings of sleep

**Figure 2:**
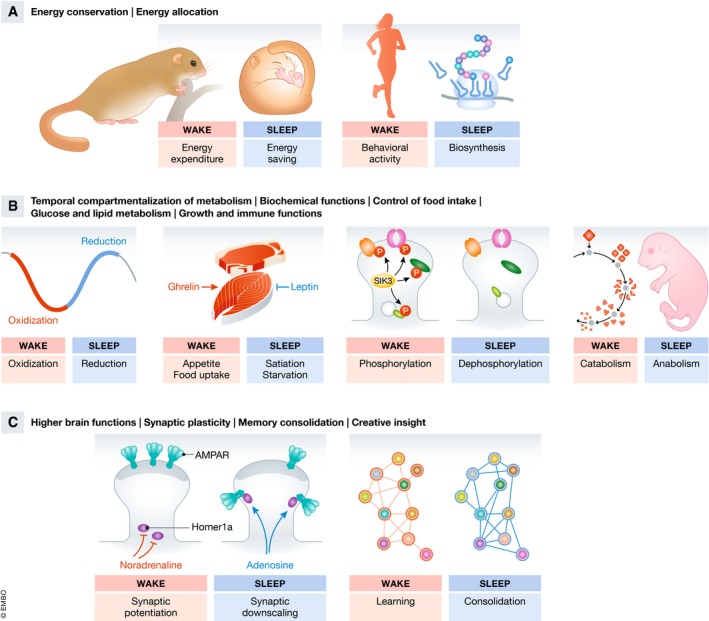
Summary of some of the hypothesized functions of sleep Various ideas exist as to the functions of sleep and molecular changes underlying sleep, and some hypotheses are depicted here. (A) In its most simple form, sleep may save energy when activity is not adaptive. It would thus serve a similar function as hibernation 22. Energy may not only be saved for later use but could rather be allocated for other processes such as anabolic reactions including protein synthesis 25. (B) Sleep may become adaptive by compartmentalizing processes such as conflicting metabolic reactions which would make these processes more efficient 36. Sleep controls hormones, food intake, and metabolism (including lipid and sugar metabolism) 3, 4. Sleep controls cyclic biochemical reactions. Wakefulness, for example, is associated with the phosphorylation of synaptic proteins and sleep is associated with dephosphorylation 37. Various other ideas as to sleep homeostasis exist, including accumulation of extracellular adenosine 144. Sleep is important for growth and immune functions 32, 33, 34. (C) Sleep controls higher brain functions such as synaptic plasticity including learning and memory. Synaptic changes during sleep include a downscaling of weak synapses, a process that appears to be promoted by Homer1a. Strong synapses are preserved 45, 47, 145. Sleep may support systems memory consolidation by re‐activating and re‐distributing memory across brain areas and circuits 49. These brain re‐arrangements may even facilitate novel insight and creativity in humans 50. Note that these ideas are overlapping. Most evidence in support of these theories stems from sleep deprivation by sensory stimulation.

The physiological state of sleep has been proposed to play multiple roles that can be coarsely sorted into three groups that are overlapping and not mutually exclusive. (i) The first group of sleep function theories posits that sleep plays a role in optimizing behavior and the conservation or allocation of energy. (ii) The second group states that sleep may regulate core molecular and cellular processes. (iii) And the third group suggests that sleep serves higher brain functions 12, 23 (Fig 2).

1. An adaptive value of sleep could be understood by viewing sleep as an inactive state. At times when wakefulness is not advantageous, the organism would enter an inactive state and thus save energy. A strong argument that energetic and ecological constraints play a role in determining sleep is the large variation in sleep amount and intensity seen across species 22. Sleep would thus share an energy‐saving function with torpor, a metabolically and behaviorally inactive phase found in mammals and birds that is characterized by a massive drop in body temperature, for instance during hibernation. Both the transitions from wakefulness to torpor as well as the exit from torpor into wakefulness involve a phase of non‐REM sleep, suggesting that they are related 22, 24, 25. Sleep and torpor differ behaviorally as sleep is defined as a readily reversible state, whereas torpor generally is not rapidly reversible. A main functional difference of torpor and sleep is that sleep need does not appear to dissipate during torpor 26, 27. Thus, sleep seems to serve benefits that cannot be simply explained by an energy conservation function alone. According to the energy allocation theory of sleep, energy is not primarily conserved for later use but is diverted to restorative processes such as anabolic biosynthetic reactions 25, 28.
2. It has been proposed that sleep becomes regenerative by allowing or facilitating key molecular and cellular housekeeping functions. This view has been supported by biochemical and transcriptomic studies that found that sleep is associated with an increase in the expression of genes required for biosynthesis and transport 29, 30, 31. Anabolic metabolism during sleep could, for example, facilitate growth, increase stress resistance, and support the immune system 32, 33, 34, 35. Sleep may control metabolism, at least in part, by regulating the rhythmic timing of food intake. For instance, sleep restriction in humans increases the concentration of the appetite‐stimulating hormone ghrelin, whereas it reduces the concentration of the appetite‐inhibiting hormone leptin, and sleep restriction is associated with metabolic syndrome, obesity, and type 2 diabetes 3, 4. Sleep may itself present a metabolic cycle, which provides a temporal compartmentalization of processes that are difficult to reconcile or that are more energetically favorable if carried out subsequently 36. An example of a cycling biochemical reaction is phosphorylation of a substantial fraction of synaptic proteins, which is globally increased during wakefulness, but decreased during sleep 37. The key kinase responsible for this phosphorylation cycle is SIK3, and a gain‐of‐function mutation of SIK3 called *sleepy* causes excessive sleep duration and intensity 38. SIK3 is a known regulator of lipid and sugar metabolism, suggesting a molecular link between sleep and cyclic metabolic activity 39, 40. Completing the picture of cellular housekeeping, it has been observed that sleep in mice is also a period in which potentially toxic molecules such as protein aggregates are removed from the brain. This removal may involve neuronal shrinkage increasing the flux of interstitial fluid 41.
3. Seminal experiments showed that a good night's sleep is important for learning and memory. Memory formation requires synaptic and cellular changes across neural circuits and brain regions that encode this memory. Transcriptome analysis of sleeping brains has found that an increased expression of genes required for plasticity and protein synthesis during sleep is required for memory formation, suggesting that sleep serves the expression of plasticity genes to support learning 42, 43, 44. Plasticity involves alterations in the size and composition of synapses. For new memories to form, specific synapses need to strengthen and new synapses need to form whereas other synapses need to weaken or disappear. It has been proposed that wakefulness leads to a net increase in synapse size and that sleep causes a subsequent net synaptic downscaling, which mostly affects weak synapses and leaves strong synapses intact 45. The weakening of synapses involves a phosphorylation and subsequent removal of AMPA receptors, a process that is supported by Homer1a 46. According to the working model, Homer1a is kept out of the synapse during wakefulness by noradrenergic signaling and enters the synapse during sleep. This recruitment of Homer1a to the synapse is promoted by adenosine, a somnogenic (sleep‐promoting) factor that is thought to accumulate as a function of wakefulness and that promotes homeostatic sleep drive 47, 48. Besides these cell biological changes of synapse size and composition, the process of memory consolidation occurs at the systems level involving recurrent reactivation of memories and its redistribution and integration into existing circuits, allowing the updating of knowledge. Disconnecting neural circuits from sensory input may facilitate the massive restructuring of brain circuits as memories mature 49. Thus, sleep may even allow novel associations and creative insights into problems that are hard to solve during wakefulness 50. REM sleep may help consolidate certain types of memories, a process that, at least in part, is mediated by rhythmic activity in the hippocampus, though the underlying mechanisms are not well understood 6, 49.

## Sleep is induced by sleep‐active neurons

The nervous system plays a crucial role in sleep induction. Research on the neural substrates of sleep control started with work on human patients who had suffered from sleep loss as a consequence of infection‐induced neural injury. Lesions in a particular brain area, the anterior hypothalamus, led to a reduction of sleep, demonstrating that dedicated centers exist in the mammalian brain that control sleep 51. This work motivated mechanistic studies of neuronal sleep control centers, mostly by using mammals such as cats, rats, and mice. Central to sleep induction are sleep‐active sleep‐promoting neurons that express inhibitory neurotransmitters, GABA, and neuropeptides. Sleep‐active neurons depolarize specifically at the onset of sleep to inhibit wake‐promoting circuits and thus to promote sleep. These neurons can be inhibited by sensory stimulation and arousal to allow quick reversibility. They are over‐activated in the process of sleep homeostasis and confer increased sleep drive. Sleep‐active neurons thus present the motor of sleep, which in turn is regulated by upstream driver mechanisms that determine when and how much the sleep motor is active 52, 53.

## Sleep deprivation reveals sleep functions

**Figure 3:**
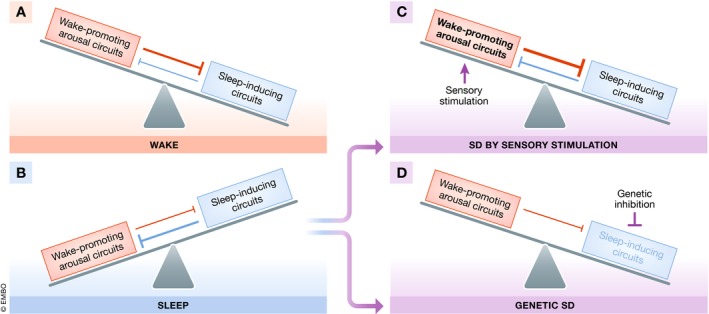
Classic SD suppresses sleep by increasing arousal, whereas genetic SD impairs the sleep‐inducing system According to the flip‐flop switch model, sleep and wake are under the control of two antagonizing systems, a wake‐inducing arousal system and a sleep‐inducing system 52. (A) During wake, the arousal system dominates and suppresses sleep. (B) During sleep, the sleep‐inducing system dominates and suppresses wake. (C) Sensory stimulation during sleep increases the activity of the arousal system, suppressing sleep despite increased sleep drive. (D) Genetically impairing the sleep‐inducing system permits wakefulness by disinhibition. Sleep‐active neurons might also contribute to arousal dampening as part of the normal waking behavior and thus their ablation might cause some level of hyperarousal. However, this arousing effect likely is smaller than the level of hyperactivity caused by sensory stimulation‐induced SD, and genetic manipulations can remove sleep without causing massive hyperactivity. Both SD approaches change the organism by fundamentally different means and are thus complementary. Both approaches should be pursued for establishing a causal link between sleep and phenotypes observed after sleep deprivation.

Most of the theories regarding the functions of sleep are based on observations of processes that correlate with sleep, and causality is established by studying the consequences of sleep deprivation. Sleep is under the control of wakefulness‐promoting and sleep‐promoting circuits, which oppose each other to generate discrete states 54. SD is typically induced by sensory stimulation, i.e., by increasing the activity of the wake‐promoting arousal system leading to an inhibition of the sleep‐promoting system. Stimulation‐induced SD accounts for virtually all the causal testing of the theories summarized above. Acute complete SD has been used to study the essential functions of sleep. Complete SD in rodents caused weight loss, skin ulceration, sepsis, and ultimately death in experimental animals 55. To prevent lethality, SD can be applied partially to shorten sleep and then is often called sleep restriction, which often is imposed chronically to study sleep functions. Chronic sleep restriction in animal models has been important to understand the effects of chronic sleep curtailment on human health. For example, sleep restriction in rodents leads to neuronal injury and reduced vigilance 56. However, it has been difficult to attribute the detrimental consequences of complete or partial SD to sleep loss rather than to stress. The pleiotropic consequences of complete SD have also made it impossible to clearly deduce the more immediate consequences of sleep loss. Sleep, arousal, and stress are intimately linked across species, and hyperarousal caused by mental stress is the main cause of insomnia in humans 2. In mammals, hyperarousal activates the HPA axis and thus sets off a physiological stress response, which maintains arousal and suppresses sleep, perpetuating a vicious cycle 57, 58. Gentler protocols are standard today and aim to arouse by motivating instead of stressing. Nevertheless, SD still is achieved by an over stimulation of sensory and arousal pathways (Fig 3) 59. A second confounding factor for studying sleep functions after SD is the interference of homeostatic sleep rebound with wake functions. SD leads to homeostatic increases in sleep pressure that can even lead to “lapses” or “microsleep” bouts that can disturb wake functions. SD in humans causes deficits in attention, working memory, and information processing 60. While it is important to study the consequences of SD on brain performance, it is difficult to understand whether the observed defects are directly caused by sleep loss or whether they are caused by homeostatic rebound mechanisms.

## Genetic sleep deprivation

An alternative strategy to SD by sensory stimulation is to render model animals sleepless by impairing the sleep‐inducing system. In this paradigm, the organism specifically lacks sleep induction, not requiring additional stimulation. The increase in arousal following sleep neuron inhibition should be attributable to a disinhibition of the wake‐promoting system (Fig 3). How can the sleep‐inducing system be impaired? While it is possible to ablate brain parts using neurosurgical methods, a more specific way to impair sleep‐inducing brain centers is through genetic targeting. Here, I thus call the use of genetics to remove sleep “genetic SD”. Genetic SD may be achieved by the deletion of sleep genes or by genetic ablation of neurons that are required for sleep induction. Complete genetic SD likely results in lethality in many systems requiring either conditional or partial approaches. Conditional genetic SD could be generated by optogenetic or chemogenetic inhibition of sleep‐active neurons as well as by inducible knockouts to create a genetic analog of SD by sensory stimulation. Alternatively, genetic SD could be induced only partially by using hypomorphic mutations to generate genetic analogs of chronic sleep restriction. In systems in which sleep loss is not imminently lethal, chronic complete SD might be a good choice to generate strong phenotypes. As an alternative to targeting sleep‐active neurons directly, manipulating neurons that are upstream or downstream of sleep‐active neurons could be employed for removing sleep. This could be achieved, for instance, by activating neurons that inhibit sleep‐active neurons or by preventing activity reduction of wake neurons that are normally inhibited by sleep‐active neurons. To complement genetic SD studies, gain‐of‐function experiments can be devised that activate the sleep‐inducing system and cause increased sleep, or “genetic sleep gain”.

Specificity of the sleep mutant phenotype is essential to link sleep loss to its consequences. However, many mutations affect sleep indirectly. For example, circadian rhythms control global physiology, and their abrogation can also result in sleep loss 61, 62. In mutants that confer a strong circadian phenotype, it will be difficult to attribute physiological phenotypes to sleep loss. Similarly, sleep loss can be caused by mutations leading to hyperactivity. However, hyperactivity also strongly affects wake behavior and causes the same problems as SD by sensory stimulation 63. The most specific sleep loss would probably be obtained by mutating genes that are specifically required for sleep induction, i.e., sleep‐active neurons and their circuits. Because sleep‐active neurons inhibit wake circuits, the removal of the sleep‐active neurons should lead to an increase in arousal. Assuming that sleep‐active neurons play only a minor role in limiting wakefulness activity but rather a prominent role in inducing sleep, their ablation may result in moderate arousal but should not result in severe hyperarousal during normal wakefulness. Consistent with this idea, mutants exist that reduce sleep without causing hyperactivity (see below). It is possible that sleep genes and neurons play roles also in other processes and that thus complete specificity of genetic SD will be difficult or impossible in some or even all systems. However, it is likely that a high degree of specificity can be achieved in most systems, which should be sufficient for studying sleep functions.

Chronic sleep restriction in humans is associated with long‐term health consequences, and model animals that genetically reduce sleep will be important tools to study the mechanisms underlying chronic sleep restriction. For studying the functions of sleep in model organisms, it may be favorable if the degree of sleep removal is high, perhaps even complete. Homeostatic compensatory processes exist that can compensate for sleep loss. For example, reduction of sleep amount in experimental models can lead to increased sleep depth during the remaining sleep time, which, at least in part, ameliorates the consequences of sleep loss. Some animals can live with little sleep, suggesting that relatively small amounts of sleep can be sufficient to fulfill sleep's essential functions 21, 52. Thus, some sleep functions may not be detectable as long as residual sleep is present and it would be advantageous to be able to ablate sleep bound. Because sleep homeostasis induces rebound sleep through over‐activation of sleep‐active neurons, the targeting of these neurons should not only allow the control of baseline sleep, but also rebound sleep 54, 64.

## Genetically removing sleep in model systems: rodents

Seminal discoveries on sleep were made using a variety of mammalian models including mice, rats, cats, and monkeys. These model animals have been pivotal in studying both non‐REM and REM sleep. The brain structures controlling sleep in mammals have turned out to be highly conserved. Its molecular amenability has made the mouse the most intensively used species for genetic sleep studies in mammals 23, 65, 66. SD by sensory stimulation has been the main method by which sleep functions have been investigated in mammals. Genetic SD is partially possible in rodent models for both REM sleep and non‐REM sleep. Forward genetic screening for sleep mutants identified a mouse mutant called *Dreamless*, a dominant mutation in a gene that encodes an ion channel required to control neural excitability, leading to a strong reduction of REM sleep but also causing defects in other rhythmic processes 38. REM sleep is induced from non‐REM sleep by GABAergic neurons in the ventral medulla of the brain stem. Inhibition of these neurons reduces REM sleep, and it has also been possible to induce REM sleep by optogenetically depolarizing these neurons 67. Thus, the *Dreamless* mutant and optogenetic induction of REM sleep present tools to investigate REM sleep functions, but such studies have not yet been published. Proving causality for REM sleep functions has been a challenge because manipulating REM sleep typically also affects non‐REM sleep 6. REM sleep is thought to be involved in specific types of memory formation and consolidation through brain activity characterized by high‐amplitude theta waves in the hippocampal EEG. To study the effects of hippocampal theta activity on memory, the activity of GABAergic MS neurons, which are required for theta activity during REM sleep but not for REM sleep itself, was optogenetically silenced during REM sleep. Silencing GABAergic MS neurons specifically during REM sleep caused defects in specific types of memory formation, providing a causal link between hippocampal theta activity during REM sleep and memory formation 68. This example shows how optogenetics can be employed for functional studies of REM sleep 6.

**Figure 4:**
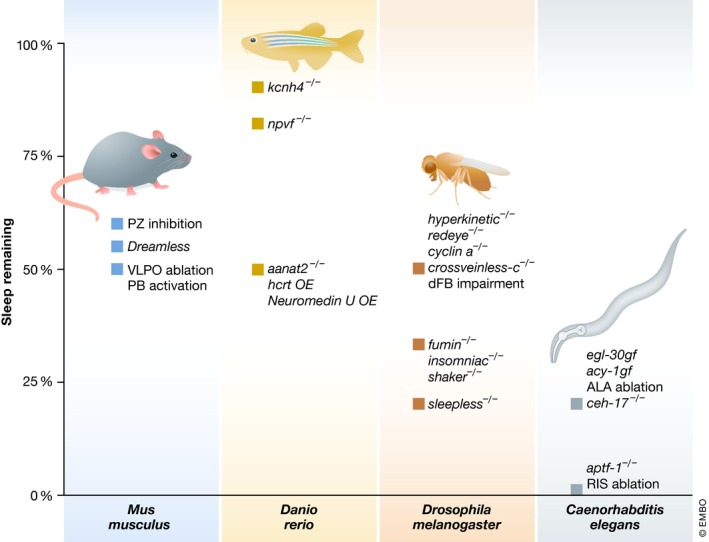
Genetic SD across the four key genetic animal models Shown are examples of genetic and neural ablations that result in reduction of sleep, some of which have low specificity, for instance manipulations leading to generally increased activity levels or impairment of circadian rhythms do not present specific manipulations. Inhibition of the PZ reduces non‐REM sleep by about 40% 77. *Dreamless* reduces REM sleep by 44% 38. Chemogenetic activation of the PB and ablation of the VLPO reduce sleep by about 50% (estimated from 70 for the first day of CNO application) 72. In zebrafish, mutation of the sleep‐promoting *npvf* gene reduces sleep by about 10% 95, voltage‐gated potassium channel gene *kcnh4a* knockout caused a 15% reduction 94, melatonin receptor mutation *aanat2* ^−/−^ led to approximately 50% reduction 96, and overexpression of wake‐promoting factors such as *hcrt* and *neuromedin u* genes led to a variable reduction of sleep of around half 90, 91. In *Drosophila*, inhibition of SIFamide receptor‐expressing PI neurons reduces sleep by about 30%(estimated from 110, 146) and interference with the mushroom body by about 45% without causing hyperactivity during wake 111. Mutation of redeye 99, cyclin A RNAi 100, deletion of hyperkinetic 101, or interference with sleep‐promoting neurons of the dFB (activating cAMP signaling 115 or *crossveinless‐c* RNAi 113) reduce sleep by about half. *insomniac* 103, *fumin* 104, or *shaker* led to approximately 2/3 reduction of sleep 102. One of the strongest mutations in *Drosophila* is *sleepless* with > 80% sleep reduction 105. *Caenorhabditis elegans* physiological sleep during lethargus is reduced by about 80% in hyperactive mutants (*egl‐30gf* 127 or *acy‐1gf* 128) as well as in ALA mutant *ceh‐17*(−) (locomotion quiescence 20 min after heat shock) 35 and is virtually abolished across several physiological conditions (reduction here displayed as 99%) by *aptf‐1* ^−/−^ or ablation of the sleep‐active RIS neuron 124, 134, 139.

**Figure 5:**
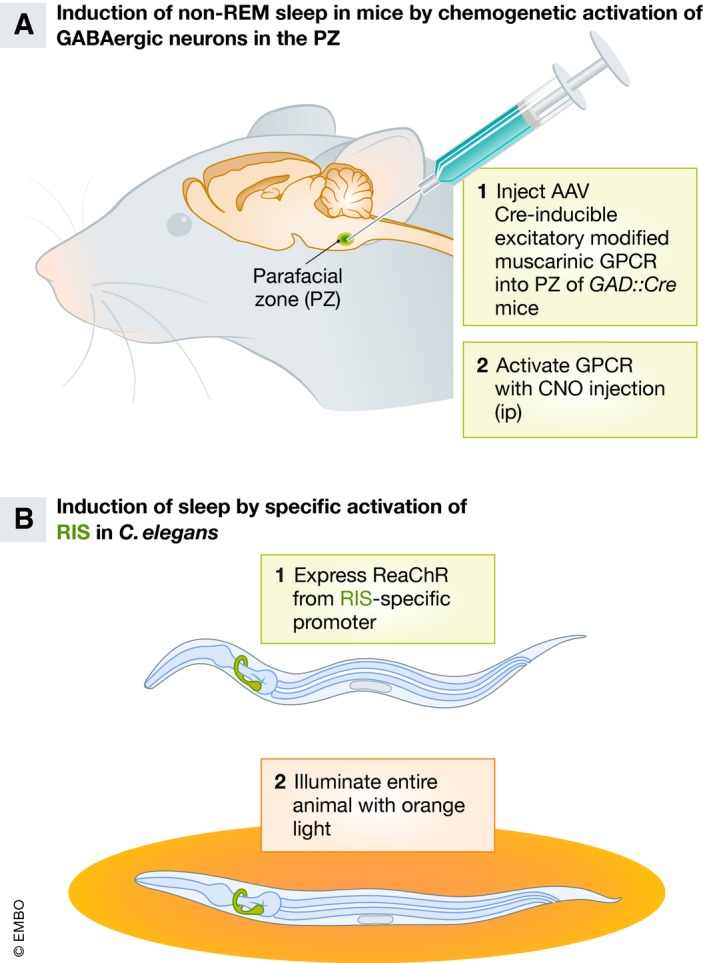
Chemogenetics and optogenetics allow specific gain‐of‐function experiments for sleep Shown are examples from mouse and *Caenorhabditis elegans*, but chemogenetic and optogenetic sleep control is also applicable to other models such as *Drosophila* and zebrafish. (A) Non‐REM sleep can be triggered in mice by chemogenetic activation of GABAergic neurons in the PZ. To achieve specific activation of GABAergic neurons within a specific brain locus, a transgenic mouse is taken that expresses Cre recombinase from the GABA‐specific GAD2 promoter. A Cre‐inducible excitatory muscarinic modified G protein‐coupled receptor is expressed using an adeno‐associated virus construct, which is injected locally into the PZ and transforms only the neurons in the vicinity of the injections. Intraperitoneal injection of CNO, an agonist of the excitatory muscarinic modified G protein‐coupled receptor, then leads to an increased activity of GABAergic PZ neurons, leading to the induction of non‐REM sleep. Mice with increased non‐REM sleep can then be analyzed for phenotypes such as learning and memory 78. (B) Sleep can be induced optogenetically in *Caenorhabditis elegans* by depolarizing the GABAergic and peptidergic sleep‐active RIS neuron 134. Transgenic animals are generated that express Channelrhodopsin (here the red‐light‐activated variant ReaChR) specifically in RIS, which is achieved by using a specific promoter. Illuminating the entire animal, which is transparent, with red light leads to the depolarization of RIS and sleep induction. The phenotypes caused by increased sleep can then be studied.

Mutants that specifically and completely remove non‐REM sleep in mammals have not yet been described, and the known mutants that show reduced sleep all display only partial sleep loss and often are not very specific but also confer additional phenotypes and are thus not ideal for genetic SD 62, 69. However, manipulations of specific brain areas can lead to substantial sleep loss or gain (Fig 4). There are two principal approaches for triggering sleep loss via manipulations of brain areas that have been successfully applied in rodents. (i) The activity of wake‐promoting areas can be increased and (ii) sleep‐inducing centers can be impaired. (i) An important wake‐promoting area is the PB, which causes arousal in many brain areas and which can be activated chemogenetically to extend wakefulness and restrict sleep for several days without causing hyperarousal 70. Alternatively to activating the PB, wakefulness can also be extended by activating other arousal centers of the brain including supramammillary glutamatergic neurons 71. (ii) Sleep‐active neurons were first found in the VLPO and lesioning this area in rodents reduced sleep by approximately 50% without causing stress, hyperarousal, or strong circadian effects 72, 73. VLPO sleep‐active neurons can also be controlled using optogenetics 74. Sleep‐promoting VLPO neurons can not only be silenced directly but also indirectly, for instance though chemogenetic activation of inhibitors of sleep‐inducing centers, such as GABAergic neurons of the ventral lateral hypothalamus or basal forebrain 75, 76. Other brain areas such as the basal forebrain, the lateral hypothalamus, brain stem, and cortex also contain sleep‐active neurons 66. For example, GABAergic neurons of the PZ of the medulla of the brainstem present an important sleep‐inducing brain region in mammals. These neurons were shown to be sleep‐active, ablation of this region led to a reduction of sleep by about 40%, and chemogenetic activation of this region led to an increase in sleep (Fig 5) 77, 78, 79. The occurrence of multiple populations of sleep‐active neurons in mammalian brains may explain why ablation of subsets of sleep‐active neurons only caused a partial removal of sleep. It would be fascinating to completely remove sleep from mice by ablating all or at least the key populations of sleep‐active neurons. A reasonable next step could be to combine genetic ablations of GABAergic sleep‐active neurons of the VLPO and PZ to see whether this would lead to a stronger or even complete sleep loss.

Restriction of sleep by VLPO lesion in rats has been used to study the role of sleep in metabolism. While ghrelin was increased and leptin was reduced, a decreased body weight and no signs of metabolic syndrome or obesity were found in this model 73. Restricting sleep by activating the PB in mice led to a modest increase in blood glucose levels and decreased leptin, but body weight was reduced similar to the results obtained by VLPO lesion 70. Interestingly, in these sleep restriction models, the metabolic consequences were thus either minor or contrary to what has been found after sleep restriction in humans 3, 4. Together these results suggest that either there are different responses of humans and rodents to sleep restriction or that the consequences of sleep restriction observed in humans may not be caused directly by sleep loss but by other factors such as stress or circadian effects, underscoring the importance to re‐evaluate sleep function theories using genetic SD models.

## Genetically removing sleep in model systems: zebrafish

The zebrafish *Danio rerio* presents an important vertebrate sleep model system between rodent and invertebrate models. Like humans and unlike rodents, zebrafish sleep mostly during the night. Zebrafish appear to have a quiet sleep state but evidence for a sleep state that resembles REM is lacking. While one study could not find evidence for rapid eye movement during sleep, this result does not exclude the possibility that other components of REM sleep are present in zebrafish 80. Major advantages of zebrafish as a sleep model are the high level of conservation of genes involved in sleep control, such as neuropeptide systems, a high level of conservation of key brain anatomical structures within a transparent brain, the possibility to model neuropsychiatric disorders as well as the possibility to scale up genetic and pharmacological screens 13, 14, 81, 82, 83, 84. Several physical methods exist for SD in zebrafish. For instance, electrical shocks and physical shaking have been used but are quite harsh and can even injure the animal 83, 85. Light potently suppresses sleep in fish leading to a 90% reduction of sleep 85. This level of sleep deprivation is impressive but sleep deprivation by light still might cause unspecific effects through sensory stimulation and alternations of the circadian clock. Perhaps the gentlest method for physical SD in zebrafish is through constant water flow 86. Physical SD in zebrafish has been mostly used to study sleep reversibility and homeostasis, but some studies have also started to address the effects of SD on cognitive functions and learning 87, 88, 89.

Through genetic screening multiple mutants with reduced sleep have been identified. For example, knockout of the sleep‐promoting neuropeptides QRFP and prokineticin 2 reduce sleep. However, these mutants produce only small effects because these factors control the relatively small amount of sleep that occurs during the day. Overexpression of wake‐promoting genes such as *hcrt* or *neuromedin U* causes hyperactivity and suppresses sleep. The effects of transient overexpression are quite variable but can suppress about half of the sleep time 90, 91. Chemogenetic or optogenetic activation or inhibition of *hcrt* neurons can be used to decrease or increase sleep, respectively 92, 93. Consistent with these findings, the *kcnh4a* potassium channel genes act in *hcrt* neurons to regulate their activity, with *kcnh4a* knockout resulting in a 15% sleep reduction 94. Loss of function of the *npvf* neuropeptide gene also causes hyperactivity and reduces sleep by 10% 95. Mutation of the melatonin receptor gene *aanat2* in zebrafish reduces night sleep in the presence of light–dark cycles by about 50%. In free‐running conditions (i.e., constant darkness), the increase of sleep during the subjective night is almost completely eliminated. These results suggest that melatonin is the major factor for circadian regulation of sleep in zebrafish 96 (Fig 4). Reports on sleep functions based on genetic SD are still lacking in the literature. While sleep‐active neurons have not yet been reported in zebrafish, they likely exist and their ablation should provide a valuable model for studying the consequences of sleep loss.

## Genetically removing sleep in model systems: *Drosophila*

*Drosophila melanogaster* has emerged as a leading model system to study the molecular basis of sleep. Its main advantages are genetic amenability and a clear coupling of sleep to the circadian rhythm. Like humans and zebrafish, *Drosophila* sleep mostly during the dark phase and also have a period of behavioral inactivity during the middle of the light phase that is called a siesta. Thus, behavioral activity in fruit flies occurs mostly during both the morning and the evening hours. *Drosophila* has been instrumental in solving the molecular underpinnings of circadian rhythms and thus presents a prime system to study the control of sleep and its regulation by the circadian clock 15, 97, 98. Genetic accessibility has motivated multiple large‐scale screens for mutations that alter sleep behavior. Mutations and neural manipulations in *Drosophila* can severely reduce sleep. For instance, mutation of the nicotinic acetylcholine receptor α subunit gene *redeye,* the potassium channel regulator *hyperkinetic,* or RNAi of *cyclin A* or its regulator reduced sleep by about half 99, 100, 101. Mutation of the *shaker* potassium channel, the ubiquitin ligase adapter complex gene *insomniac*, and the dopamine transporter gene *fumin* reduced sleep by about two‐thirds 102, 103, 104. Among the strongest mutations that reduce sleep is the *sleepless* mutation with about 80% of sleep reduction. *sleepless* encodes a neurotoxin that regulates *shaker* 105, 106 (Fig 4). However, several of these mutants are severely hyperactive. Thus, results regarding sleep functions based on hyperactive mutants should be interpreted with caution 101, 104, 105, 107.

Fly brains possess several centers that contain wake‐promoting or sleep‐promoting neurons. Wake‐promoting centers are, for example, cyclin A‐expressing neurons of the pars lateralis 108. Important sleep‐promoting centers are formed by sub‐populations of neurons in the mushroom body, dorsal paired medial neurons, and peptidergic neurons in the PI 109, 110, 111. As another example, sleep‐promoting neurons of the dFB can actively induce sleep and confer homeostatic sleep drive stemming from R2 neurons of the ellipsoid body and are thus similar to mammalian sleep‐promoting neurons 112, 113, 114. Interference with the function of dFB neurons, for instance by RNAi of *crossveinless‐c*, a Rho GTPase‐activating gene, reduced sleep by about half. Importantly, mutation of *crossveinless‐c* decreases sleep without causing signs of hyperactivity 113, 115. This supports the hypothesis that genetic SD without hyperactivity is possible in *Drosophila* (Fig 4). Thus, specific interference of dFB neurons and *crossveinless‐c* mutants present specific, albeit partial, genetic SD in *Drosophila* and should, along with other mutants, provide useful models for studying the effects of sleep restriction in fruit flies. Similar to mammals, several populations of sleep‐promoting neurons exist and the ablation of individual populations causes partial sleep loss. It is not well understood how the various sleep centers in *Drosophila* interact to cause sleep, but they likely act, at least in part, in parallel pathways. It might be possible to combine mutations that target different sleep‐promoting areas and test whether this would result in near‐complete sleep loss. This would not only shed light on how the different sleep centers interact but might also generate stronger models of genetic SD. It will be interesting to see whether near‐complete genetic SD will be possible and whether and how it would result in lethality.

Sensory stimulation‐induced SD leads to hyperarousal, the activation of cellular stress responses in *Drosophila,* and is detrimental 116. Genetic sleep reduction has been associated with reduced lifespan in many but not all *Drosophila* sleep mutants. For instance, loss of the *sleepless* gene causes both a shortening of sleep and lifespan, while neuronal knockdown of *insomniac* leads to sleep reduction without a shortening of longevity 102, 103, 105, 117. Also, knockout of *fumin* did not cause a shortening of lifespan but a reduction of brood size 104, 118. Also, defects in memory have been observed in sleep mutants 101. Genetic sleep reduction by neuronal knockdown of *insomniac* did not demonstrate a role for sleep in survival of infection or starvation. The short‐sleeping mutant did, however, exhibit a sensitivity to survive oxidative stress. Several other short‐sleeping mutants showed oxidative stress sensitivity as well, suggesting that the sensitivity was probably not conferred by pleiotropic side effects caused by the mutation but rather is broadly associated with sleep loss. Consistent with this finding, increasing sleep genetically or pharmacologically conferred greater resistance to oxidative stress 107. These experiments not only identified resistance to oxidative stress as a potential core function of sleep in *Drosophila*, but also illustrate how the use of multiple sleep mutants distills a sleep phenotype from potentially pleiotropic mutations.

## Genetically removing sleep in model systems: *C. elegans*

*Caenorhabditis elegans* is the genetic animal model with the smallest nervous system, as it has only about 0.3% the number of neurons of an adult *Drosophila* or zebrafish embryo brain. The connectome of the 302 neurons of the hermaphrodite has been mapped, providing an entry point for circuit studies 119. Sleep in *C. elegans* is attractive to study due to its genetic amenability and the invariant number of neurons allowing straightforward genetic SD. *Caenorhabditis elegans* shows sleeping behavior across many life stages and conditions. In the developing larva, sleep is linked to the molting cycle, and sleep bouts occur during a phase called lethargus prior to the molt 120, 121, 122. This developmentally controlled sleep does not seem to be coupled to the day–night cycle, but its timing still is controlled by the circadian period homolog *lin‐42* 123. If hatched in the absence of food, larvae arrest development and during this phase alternate between sleep and wake cycles 124. In the presence of adverse conditions, worms develop into an enduring alternative larval stage called the “dauer”, which spends much of its time sleeping 121, 124. Adult worms sleep both in the presence and in the absence of food, with food amount and quality determining the amount of sleep 124, 125, 126. Finally, *C. elegans* sleep following severe cellular stress 35.

As in other species, hyperactive mutations can reduce sleep in *C. elegans;* however, they do not present specific manipulations 127, 128. *Caenorhabditis elegans* possess two major individual neurons that have been implicated in the induction of sleep. Cellular stress causes the secretion of EGF, which activates EGF receptor signaling in a neuron called ALA 35, 129, 130. EGF activation leads to the secretion of multiple neuropeptides from ALA, which have both overlapping and distinct inhibitory functions on behavioral activity by binding to downstream receptors, likely involving a diffusional mechanism 131, 132, 133. It is not yet clear whether ALA presents a sleep‐active neuron in the sense that it depolarizes specifically during a sleep bout or whether it promotes sleep by a different mechanism. ALA can be easily ablated physically or genetically. Loss of function of the homeobox transcription factor genes *ceh‐17* or *ceh‐14* renders ALA dysfunctional and thus strongly impairs sleep following cellular stress 129 (Fig 4).

The second major known sleep‐promoting neuron of *C. elegans* is called RIS. This neuron is sleep‐active as it depolarizes at the onset of sleep bouts and its optogenetic depolarization acutely induces sleep 134, 135, 136 (Fig 5). Similar to ALA, RIS can be easily ablated physically or genetically. A mutation in the AP2 transcription factor gene *aptf‐1* renders RIS inactive, because AP2 is required for the expression of sleep‐inducing neuropeptides 134. Interestingly, AP2 transcription factors are conserved regulators of sleep also in *Drosophila* and humans 137, 138. Sleep bouts become undetectable in these “RIS mutants” during many life stages and physiological conditions. *aptf‐1* mutant worms show no severe hyperactivity during wake, indicating that they are not strongly hyperaroused following sleep loss and that sleep loss is likely not a consequence of increased arousal 124, 134, 135, 139. Thus, during many physiological conditions, RIS inactivation in *C. elegans* presents both a virtually complete as well as a highly specific model for sleeplessness (Fig 4). It has been proposed that ALA and RIS present mostly parallel systems that act during un‐physiological and physiological conditions, respectively, and whether and how these neurons interact is not known 140. Together, ALA and RIS ablation present valuable tools for studying the functions of sleep in different conditions.

Loss of ALA function is viable during physiological conditions but impairs survival upon cellular stress, demonstrating the importance of sleep in recuperating from cellular insult. The need to sleep after cellular stress is plastic and is reduced if the general stress resistance is increased, suggesting that sleep is part of a stress resistance program 35, 129, 130, 141. RIS‐ablated *C. elegans* are viable and display much less severe consequences compared with SD by sensory stimulation, which can even be lethal 134, 139, 142, 143. It is possible that sensory stimulation causes non‐specific side effects or that long‐term genetic SD is compensated for by development or other homeostatic processes. *Caenorhabditis elegans* lives a boom‐and‐bust lifestyle and alternates between short periods of superfluous food and long periods of starvation. Consistent with these ecological constraints, sleep becomes essential during larval starvation‐induced developmental arrest. It was shown that genetic SD shortens the survival of starved larvae by half. Rather than merely conserving energy, sleep appears to slow down the progression of aging phenotypes 124. RIS‐ablated worms have a normal lifespan under ideal laboratory conditions 124, but sleep during old age has not been examined. *Caenorhabditis elegans* has a short generation time and life span and may thus be uniquely able to survive sleep loss under ideal laboratory conditions. In summary, similar to *Drosophila*, essential sleep functions following genetic SD in *C. elegans* are most obvious under challenging conditions.

## Conclusion

Recent advances in the understanding of how sleep is regulated in genetically accessible model organisms have made it possible to genetically remove sleep to a high degree and specificity. Acute SD by sensory deprivation and chronic genetic SD are obviously different experiments and can lead to different conclusions as to the functions of sleep. In future studies, it will be important to understand the basis of these differences. It is as of now unclear whether SD by sensory stimulation overestimates the role of sleep because it causes non‐specific side effects or whether genetic SD underestimates the role of sleep because of compensation processes. Genetic SD models can be used to study the consequences of sleep restriction or loss. Until now, specific phenotypes from genetic SD are scarce. However, sleepless model animals are increasingly employed for studies aiming to understand the consequences of sleep loss and will likely be key to comprehend why animals and humans need to sleep. Initial results indicate that much of the phenotypes observed after SD may not be a direct consequence of the lost sleep. For instance, the metabolic consequences of sleep loss in humans have been challenged by more specific surgical or genetic SD in rodents. Similarly, genetic SD in *Drosophila* and *C. elegans* produces smaller phenotypes compared with stimulation‐induced SD. Work from *Drosophila* and *C. elegans* suggests that sleep becomes especially important for survival during challenging conditions. Improving the genetic sleep loss models by increasing the degree and specificity of sleeplessness as well as fine‐tuning the amount and timing of lost or gained sleep will be important next steps in facilitating the study of sleep functions in animals. Analyzing phenotypes of genetic SD models will help define core functions of sleep and support our endeavor to understand how sleep becomes vital.

## Conflict of interest

The author declares that he has no conflict of interest.

**In need of answers**

- What are the vital functions of sleep?
  The functions of sleep have been studied for decades, mostly by either correlation or SD induced by sensory stimulation. Genetic SD is an emerging alternative to remove sleep but typically produces weaker phenotypes compared with stimulation‐induced SD. It could be that constitutive genetic SD leads to compensatory changes, whereas acute SD cannot be easily compensated for. However, the power of constitutive genetic SD lies in the potential accumulation of the consequences of sleep loss over time. Also, transgenerational effects of sleep loss should be studied for long‐term effects of sleep loss. Thus, a thorough analysis of the different SD methods and a re‐evaluation of the previously proposed roles of sleep will be necessary to understand sleep functions.
- Can sleep be removed specifically and completely using genetic SD?
  A prerequisite for genetic SD is specificity of the manipulation as well as a high degree of deprivation. However, it is yet unclear what level of specificity can be achieved. Genes and neurons that control sleep may have functions that overlap with other processes. Also, complete genetic SD likely is lethal in many systems such as mammals. Thus, partial or conditional genetic SD will be the methods of choice for studying sleep functions in this case.
- How did sleep evolve and how conserved are sleep functions?
  Molecular analysis has suggested that there is a high level of conservation of sleep regulation but it is less clear how conserved molecular sleep functions are. Also, it is not clear for which initial functions sleep has been selected for. Speculatively, sleep emerged in evolution to save energy or serve basic needs and was co‐opted later to also serve higher brain functions. Studying sleep functions across models should shed light on these questions. Evolutionary studies would be aided by studying sleep and sleeplessness in additional models beyond the widely used animals described herein.
- How does sleep exert its functions?
  While many ideas exist as to the potential functions of sleep, little is known about the underlying mechanisms. For example, it is not known what molecules are preserved, how resources are allocated, and how cellular processes are aided. It is unclear how basic molecular and cellular functions of sleep support a healthy physiology and how sleep is linked to aging. Also, how sleep aids higher brain functions is not clear. To answer these questions, the consequences of sleep loss need to be studied using multiple molecular and systems approaches across animals.
